# Prediction of inherited metabolic disorders using tandem mass spectrometry data with the help of artificial neural networks

**DOI:** 10.55730/1300-0144.5840

**Published:** 2024-07-12

**Authors:** Pembe SOYLU ÜSTKOYUNCU, Nurettin ÜSTKOYUNCU

**Affiliations:** 1 Division of Pediatric Nutrition and Metabolism, Department of Pediatrics, Faculty of Medicine, Health Sciences University, Kayseri, Turkiye; 2 Department of Electrical & Electronics Engineering, Faculty of Engineering, Erciyes University, Kayseri, Turkiye

**Keywords:** Artificial intelligence, artificial neural networks, inborn errors of metabolism, children, prediction

## Abstract

**Background/aim:**

Tandem mass spectrometry is helpful in diagnosing amino acid metabolism disorders, organic acidemias, and fatty acid oxidation disorders and can provide rapid and accurate diagnosis for inborn errors of metabolism. The aim of this study was to predict inborn errors of metabolism in children with the help of artificial neural networks using tandem mass spectrometry data.

**Materials and methods:**

Forty-seven and 13 parameters of tandem mass spectrometry datasets obtained from 2938 different patients were respectively taken into account to train and test the artificial neural networks. Different artificial neural network models were established to obtain better prediction performances. The obtained results were compared with each other for fair comparisons.

**Results:**

The best results were obtained by using the rectified linear unit activation function. One, two, and three hidden layers were considered for artificial neural network models established with both 47 and 13 parameters. The sensitivity of model B2 for definitive inherited metabolic disorders was found to be 80%. The accuracy rates of model A3 and model B2 are 99.3% and 99.2%, respectively. The area under the curve value of model A3 was 0.87, while that of model B2 was 0.90.

**Conclusion:**

The results showed that the proposed artificial neural networks are capable of predicting inborn errors of metabolism very accurately. Therefore, developing new technologies to identify and predict inborn errors of metabolism will be very useful.

## 1. Introduction

Inborn errors of metabolism are heterogeneous disorders resulting from defects in biochemical pathways. These disorders are individually rare but account for a significant portion of childhood disability and deaths. Hundreds of disorders have been described to date. They can manifest over a wide period of time, starting from the intrauterine period and continuing to adulthood [1].

Tandem mass spectrometry (MS) has changed our ability to detect intermediates of metabolism in small samples and makes it possible to detect large numbers of metabolic disorders in a single analysis. It is used for screening, diagnosis, and disease monitoring. Over 60 different metabolic disorders can be screened by tandem MS. It is helpful in diagnosing amino acid metabolism disorders, organic acidemias, and fatty acid oxidation disorders, and it can provide rapid and accurate diagnoses for inborn errors of metabolism [2–8].

Artificial intelligence (AI) techniques have been used to support clinical decision-making processes since the introduction of computer technology [9,10]. Many different classical, AI, and machine learning techniques such as artificial neural networks (ANNs), naive Bayes classifiers, support vector machines (SVMs), and decision trees have been used for the prediction and classification of medical diagnoses. ANNs have been used in many different areas such as engineering, finance, and medicine in recent decades [11,12]. They are very good solutions for predicting diagnoses. They can be used with complex clinical datasets to predict complex and nonlinear relationships [13,14]. ANNs are structured based on biological neurons and they have learning and generalization abilities. They can provide better performance compared to classical statistical methods. ANNs use multiple layers of calculations to imitate the ways in which the human brain interprets and draws conclusions from information.

The aim of this study was to predict inborn errors of metabolism in children with the help of ANNs using tandem MS data.

## 2. Materials and methods

### 2.1. Data selection

Tandem MS data obtained from 2938 different individuals at one time in the Health Sciences University Kayseri City Hospital between July 2018 and December 2022 were evaluated retrospectively. The data were divided into two groups as suspected inherited metabolic disorders (SIMDs) and definitive inherited metabolic disorders (DIMDs). There were 2893 tandem MS datasets for the SIMD group and 45 tandem MS datasets for the DIMD group. The datasets used for the ANNs are shown in Table 1.

### 2.2. Parameter selection

All 47 parameters in the tandem MS datasets were used for the training and testing of models A. The number of parameters was then reduced to 13 by using statistical methods and expert knowledge. We achieved simpler ANN structures and the need for computational effort was decreased by reducing the parameters. The 13 selected parameters were used for the training and testing of models B. The parameters used in the diagnosis of inherited metabolic disorders are shown in Tables 2 and 3.

### 2.3. Statistical analysis

Statistical evaluation was performed with SPSS (SPSS Inc., Chicago, IL, USA). Histograms, q-q graphs, and Shapiro–Wilk normality tests were used to examine whether the data showed normal distribution. Abnormally distributed parameters were expressed as medians and 25th–75th percentiles. The 47 parameters of tandem MS were compared statistically between the two groups. The Mann–Whitney U test was performed for parameters that were not normally distributed variables. Values of p < 0.05 were considered statistically significant in all statistical analyses. Statistical evaluation of the datasets is shown in Table 3. Univariate logistic regression analysis of the datasets is shown in Table 4.

### 2.4. Artificial intelligence model

**Figure 1:**
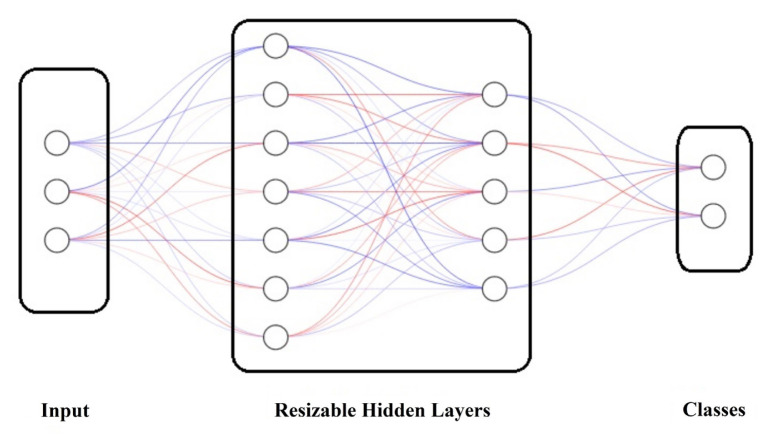
General structure of the ANN classifier.

MATLAB software was used for the ANN studies. All ANN models used in this study for classification were feedforward and fully connected (FC) neural networks. The general structure of a neural classifier is shown in Figure 1. The neural classifiers used in this study had fully connected/hidden layers. The first hidden layer of the ANN had a connection to the input. An activation function such as rectified linear unit (ReLU), hyperbolic tangent, or sigmoid function was applied to each FC layer except the last layer. The softmax transfer function was applied to the last FC layer to produce the network’s output and the output layer corresponded to the predicted classes. The data were divided into two groups randomly to be used in training and testing the ANNs. While 75% of the dataset was used for training, 25% was used for testing. The datasets used for the ANNs are shown in Table 1, as mentioned above. After the ANN structures were trained with the training dataset containing all parameters, testing was carried out using the testing data. The number of parameters was then decreased to 13 and all processes were repeated. ANN structures with different numbers of hidden layers and neurons were established to obtain better results with less computational effort and with fewer neuron numbers in the layers. One, two, and three hidden layers were taken into account for the ANN models obtained with both 47 and 13 parameters. The neuron numbers of each layer were limited to 50 neurons, and the ANN models with fewer neurons and the same results are the ones presented in this paper.

### 2.5. Ethical approval

The study was conducted in accordance with the Declaration of Helsinki and good clinical practice ethics. It was approved by the local ethics committee of Kayseri City Hospital (Number: 911/2023).

## 3. Results

Forty-seven and 13 selected parameters of tandem MS datasets from 2938 different patients at one time were taken into account to train and test the ANNs. There were 2893 datasets for the SIMD group and 45 datasets for the DIMD group (Table 1).

C3, C4, C5, C50H, C5DC, C6, C10:1, C12, arginine, leucine, citrulline, phenylalanine, and glycine were used in the diagnosis of inherited metabolic disorders, as shown in Table 2.

The 47 parameters of tandem MS were compared statistically between the two groups. Mann–Whitney U tests were performed for parameters that were not normally distributed variables, as shown in Table 3. Univariate logistic regression analysis was performed for parameters that were statistically significant in the Mann–Whitney U tests and selected for the ANNs. C4, C5, C50H, phenylalanine, and glycine were found to be statistically significant and positively correlated with DIMDs in logistic regression analysis. The results of the univariate logistic regression analysis of the datasets are shown in Table 4.

Only the results of the ANN models with the ReLU activation function are given in this study because the best results were obtained using this activation function. All 47 parameters of tandem MS were used for the training and testing of models A. Thirteen selected parameters were used for the training and testing of models B. Model A3 and Model B2 were found to be the most effective models in predicting DIMDs. Model B2 could not correctly predict the data of patients with multiple acyl-CoA dehydrogenase deficiency, glutaric aciduria type-1, and nonketotic hyperglycinemia. The best three ANN models with 47 parameters and their prediction results and the best three ANN models with 13 parameters and their prediction results are shown in Tables 5 and 6, respectively.

The highest accuracy rates were detected for models A3 and B2. The accuracy rate of model A3 was 99.3% and the accuracy rate of model B2 was 99.2%. The area under the curve (AUC) value of model A3 for DIMDs was 0.87, and the AUC value of model B2 for DIMDs was 0.90. Test accuracy and AUC values of the ANNs are shown in Table 7.

**Figure 2:**
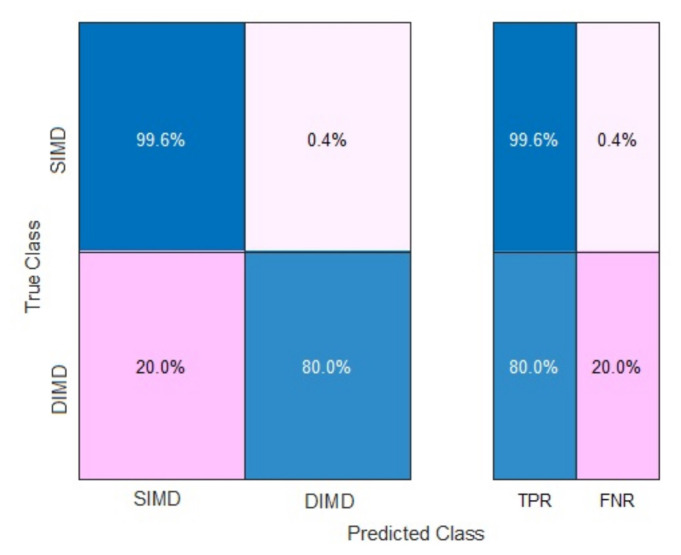
TPR and FNR tables for the testing of model B2-ANN. SIMD: Suspected inherited metabolic disorder; DIMD: definitive inherited metabolic disorder; TPR: true positive rate, FNR: false negative rate.

The sensitivity of test model B2-ANN was found to be 80%. True positive rates (TPRs) and false negative rates (FNRs) of the testing for model B2-ANN are shown in Figure 2.

## 4. Discussion

There are few studies evaluating inherited metabolic disorders with the use of AI. Studies on this subject have mostly focused on newborn screening programs. Different machine learning methods have been applied to support newborn screening programs. Most studies only focus on a single disease or specific machine learning techniques, making it difficult to conclude which methods are best to implement [15–19].

Baumgartner et al. [20] reported that they used six machine learning techniques for newborn screening by tandem MS. An ANN was among the machine learning techniques in this study, entailing a multilayered ANN trained using backpropagation. They reported the accuracy rates for two inherited metabolic disorders, phenylketonuria and medium-chain acyl-CoA dehydrogenase deficiency. The accuracy rate of the ANN was 99.2% for phenylketonuria and 99.3% for medium-chain acyl-CoA dehydrogenase deficiency [20]. The ANN was one of the most powerful machine learning techniques for predicting two specific inherited metabolic disorders in that study. Although our ANNs evaluated more than one parameter and more than one inherited metabolic disorder, similar prediction rates were detected in our study.

Hsu et al. [21] reported that the prediction accuracy for methylmalonic acidemia could be improved from 56%–73% to over 96% and the sensitivity could be improved from 70%–81% to over 95% after applying a modified SVM classifier in a newborn screening program [21]. The TPR of test model B2-ANN was found to be 80% and the FNR was 20% for DIMD in our study. This ANN failed to predict three inherited metabolic disorders correctly in our study. Increasing the amount of DIMDs in the datasets could improve the predictive performance of ANN models.

Peng et al. reported that random forest-based analysis reduced the FPRs for glutaric acidemia type-1 by 89% and for ornithine transcarbamylase deficiency by 98% [22]. Zaunseder et al. [23] reported that logistic regression analysis (LRA) was interpretable on a modular level and more applicable for newborn screening. They concluded that noninterpretable methods such as Ridge-LRA and Bagging-SVM showed promising results. Although several machine learning techniques have been used in different studies, these methods do not have a clear advantage over each other.

Apart from newborn screening, AI has also been used in specific metabolic diseases such as Fabry disease, Pompe disease, and alkaptonuria. Jefferies et al. [24] analyzed the performance of AI in identifying patients with Fabry disease. AI was calibrated by using health record data from a large cohort of 5000 patients with Fabry disease, and phenotypic patterns were extracted from those records. The study dataset was divided into a training set comprising 75% of all patients selected at random and a testing cohort comprising the remaining 25%. AI demonstrated strong analytical performance in identifying patients with Fabry disease. The AUC value of the test was 0.82 in that study. That study is similar to our study in some regards. The results of our study show that the established ANNs are capable of predicting inborn errors of metabolism very accurately. The AUC of the test for model B2 in our study was 0.90.

Wilkes et al. [25] developed decision support classifiers with several machine learning algorithms using 2084 plasma amino acid data. They tested the generalization performance of each classifier using a nested cross-validation procedure. The classifiers demonstrated excellent predictive performance, with the three machine learning algorithms tested producing comparable results. The best-performing classifier achieved mean precision-recall with an AUC of 0.957. Twelve amino acids and a total of 35,256 data (12 × 2938) belonging to those amino acids were evaluated with a different AI technique in our study. The AUC value of the most successful ANN model was determined as 0.90.

Models A3 and B2 were considered superior to other models in our study because they predicted DIMDs with less error than the other models. Although the sensitivity of model B2 was found to be 80%, this model could not correctly predict the data of the patients with multiple acyl-CoA dehydrogenase deficiency, glutaric aciduria type-1, or nonketotic hyperglycinemia in our study. This can be explained by the fact that the glycine levels in nonketotic hyperglycinemia and the C5DC levels in glutaric aciduria type-1 are very close to the reference values.

The main limitation of our study is that the amount of data belonging to children with inherited metabolic disorders is very limited because DIMDs have low incidence rates. Increasing the amount of DIMDs included in the datasets could improve the predictive performance of ANN models. We anticipate that AI studies will help doctors working in the field of pediatric metabolism.

In conclusion, the diagnosis of inborn errors of metabolism currently requires expert knowledge. Developing new technologies to identify and predict inborn errors of metabolism will be very useful. Inborn errors of metabolism were predicted with the use of ANNs in this study. Tandem MS results of 2938 children were used for ANNs to predict inborn errors of metabolism. The ANN approaches were compared with each other to show the differences between them. The highest accuracy rates were detected for models A3 and B2. The sensitivity of model B2 was found to be 80%. The results showed that the established ANNs are capable of predicting inborn errors of metabolism very accurately.

## Figures and Tables

**Table 1:** Datasets used for ANNs.

Process	Datasets	SIMD	DIMD
Training	2203	2173	30
Test	735	720	15
Total	2938	2893	45

SIMD: Suspected inherited metabolic disorder; DIMD: definitive inherited metabolic disorder.

**Table 2:** Parameters used in the diagnosis of inherited metabolic disorders.

Parameter	Inborn errors of metabolism
C3	MMA, PA, disorders of cobalamin synthesis
C4	MADD
C5	IVA, MADD
C5OH	MCC deficiency, HMG-CoA lyase deficiency, HCLS deficiency
C5DC	GA-1, MADD
C6	MADD
C10:1	MADD
C12	MADD
Arginine	Argininemia
Leucine	MSUD
Citrulline	CTLN1
Phenylalanine	PKU, HFA
Glycine	NKH, MMA, PA

MMA: Methylmalonic acidemia; PA: propionic acidemia; IVA: isovaleric acidemia; MADD: multiple acyl-CoA dehydrogenase deficiency; MCC: 3-methylcrotonyl CoA carboxylase deficiency; HMG-CoA lyase deficiency: 3-hydroxy-3-methylglutaryl-coenzyme A lyase deficiency; HCLS deficiency: holocarboxylase synthetase deficiency; GA-1: glutaric aciduria type-1; MSUD: maple syrup urine disease; CTLN1: citrullinemia type-1; PKU: phenylketonuria; HFA: hyperphenylalaninemia; NKH: nonketotic hyperglycinemia.

**Table 3:** Statistical evaluation of the data and parameter selection (Mann–Whitney U tests).

Number	Parameters	Selected parameters	SIMD median (25th–75th percentiles, %)	DIMD median (25th–75th percentiles, %)	p
1	C6DC		0.01 (0.01–0.04)	0.02 (0.01–0.07)	0.020
2	C18:1 OH		0.01 (0–0.1)	0.01 (0–0.01)	0.576
3	C2		13.95 (9.5–20.2)	16.4 (10–26.2)	0.129
4	C4	X	0.18 (0.12–0.26)	0.23 (0.14–0.38)	**0.042**
5	C16OH		0.01 (0–0.03)	0.01 (0–0.031)	0.961
6	C18:2		0.03 (0.02–0.08)	0.04 (0.02–0.11)	0.256
7	C3/C0		0.05 (0.03–0.06)	0.05 (0.03–0.12)	0.260
8	C3/C2		0.09 (0.06–0.13)	0.1 (0.05–0.18)	0.509
9	C3DC		0.02 (0.01–0.03)	0.01 (0.01–0.03)	0.581
10	C4OH		0.02 (0.01–0.06)	0.04 (0.02–0.18)	0.000
11	C5OH	X	0.09 (0.05–0.14)	0.13 (0.06–0.33)	**0.001**
12	C6	X	0.06 (0.04–0.09)	0.07 (0.05–0.1)	**0.030**
13	C8/C10		0.8 (0.5–1)	1 (0.71–1.5)	0.003
14	C10		0.05 (0.03–0.08)	0.05 (0.02–0.08)	0.730
15	C10:1	X	0.03 (0.01–0.05	0.04 (0.02–0.07)	**0.011**
16	C12	X	0.05 (0.03–0.07)	0.05 (0.03–0.1)	0.205
17	C5DC	X	0.06 (0.03–0.09)	0.07 (0.03–0.1)	0.156
18	C5	X	0.11 (0.08–0.17)	0.17 (0.11–0.34)	**0.000**
19	Methyl-glutaryl		0.02 (0–0.03)	0.02 (0.01–0.04)	0.121
20	C4DC		0.12 (0.08–0.18)	0.12 (0.09–0.17)	0.814
21	C14		0.06 (0.03–0.09)	0.08 (0.03–0.11)	0.085
22	C8		0.04 (0.02–0.06)	0.05 (0.02–0.08)	0.057
23	C8:1		0.03 (0.01–0.06)	0.03 (0.01–0.05)	0.601
24	C18:1		0.11 (0.07–0.2)	0.12 (0.07–0.29)	0.179
25	C16		0.53 (0.31–0.78)	0.63 (0.35–1.2)	0.058
26	C16:1		0.01 (0–0.02)	0.02 (0–0.04)	0.013
27	Phe/Tyr		0.62 (0.45–0.84)	0.77 (0.5–0.75)	0.017
28	C3	X	1.3 (0.88–1.89)	1.5 (0.75–3.79)	0.240
29	C10DC		0.01 (0–0.03)	0.01 (0.005–0.03)	0.570
30	C0		28.6 (22.35–36.58)	30.8 (21.9–39.6)	0.476
31	C18		0.26 (0.16–0.38)	0.34 (0.16–0.49)	0.057
32	C8DC		0.01 (0–0.03)	0.01 (0.01–0.05)	0.693
33	C14:2		0.02 (0–0.04)	0.02 (0.01–0.04)	0.051
34	C14:1		0.02 (0.01–0.04)	0.02 (0.01–0.08)	0.030
35	C5:1		0.02 (0.01–0.05)	0.02 (0.01–0.055)	0.508
36	Alanine		293.5 (226.99–375.67)	340.7 (235.1–391.1)	0.104
37	Arginine	X	33.1 (20.69–50.6)	33.5 (20.3–56.2)	0.756
38	Aspartate		51.8 (38.16–70.06)	56.5 (40.8–82.5)	0.153
39	Phenylalanine	X	46.67 (36.25–59.58)	49.3 (41.1–62.0)	**0.041**
40	Glycine	X	246.87 (194.03–330.29)	269 (210.2–447)	**0.013**
41	Glutamate		135.2 (100.54–180.44)	135.6 (71.7–190.7)	0.831
42	Glutamine		122.5 (69.64–190.52)	127.4 (86.5–248.2)	0.067
43	Leucine	X	108.1 (83.93–137.14)	110 (84.4–160.0)	0.264
44	Methionine		18.8 (13.9–25.66)	18.7 (15.2–25.3)	0.822
45	Citrulline	X	21.4 (15.66–28.04)	26.4 (14.2–35.6)	0.099
46	Tyrosine		74.9 (56.42–101.98)	65.6 (48.9–103.7)	0.337
47	Valine		123.4 (95.81–156.53)	129.1 (101.6–159.5)	0.293

SIMD: Suspected inherited metabolic disorder; DIMD: definitive inherited metabolic disorder. Significant p-values are shown in bold.

**Table 4:** Univariate logistic regression analysis of data.

Univariate analysis			
	OR	95% CI	p
C4	4.575	1.446–14.47	0.010
C5	373.2	27.01–5116	0.000
C5OH	47.82	6.966–328.3	0.000
Phenylalanine	1.014	1.008–1.020	0.000
Glycine	1.002	1.001–1.004	0.000

CI: Confidence interval; OR: odds ratio.

**Table 5:** The best three ANN models with 47 parameters and their prediction results.

Hidden layer numbers and model name	Neuron numbers	Training prediction results				Testing prediction results			
		SIMD		DIMD		SIMD		DIMD	
		True	False	True	False	True	False	True	False
1 & Model A1	18	2173	-	30	-	717	3	11	4
2 & Model A2	32–11	2173	-	30	-	718	2	10	5
3 & Model A3	14–9–4	2173	-	30	-	719	1	11	4

SIMD: Suspected inherited metabolic disorder; DIMD: definitive inherited metabolic disorder.

**Table 6:** The best three ANN structures with 13 parameters and their prediction results.

Hidden layer numbers and model name	Neuron numbers	Training prediction results				Testing prediction results			
		SIMD		DIMD		SIMD		DIMD	
		True	False	True	False	True	False	True	False
1 & Model B1	3	2172	1	27	3	714	6	12	3
2 & Model B2	27–3	2171	2	30	-	717	3	12	3
3 & Model B3	8–4–8	2171	2	30	-	712	8	13	2

SIMD: Suspected inherited metabolic disorder; DIMD: definitive inherited metabolic disorder.

**Table 7:** Testing accuracy and AUC values of ANNs.

	Model A1	Model A2	Model A3	Model B1	Model B2	Model B3
Accuracy	0.9905	0.9905	0.9932	0.9878	0.9918	0.9864
AUC (DIMD)	0.8646	0.8319	0.8660	0.8958	0.8979	0.9278

AUC: Area under the curve; DIMD: definitive inherited metabolic disorder.
